# Supplementary material for: A methodology to reduce the localization error in multi-loci microscopy provides new insights into enhancer biology
Source: PLoS Comput Biol. 2025 Dec 17;21(12):e1013796. doi: 10.1371/journal.pcbi.1013796 (PMC12747437; doi:10.1371/journal.pcbi.1013796)
Supplement: Attachment — Submitted filename: Resp_To_Reviewers_PCOMPBIOL.pdf [file pcbi.1013796.s002.pdf]

PCOMPBIOL-D-25-00741

A methodology to reduce the localization error in multi-loci microscopy provides new insights into enhancer biology

PLOS Computational Biology

Dear Dr. Larson,

Thank you for submitting your manuscript to PLOS Computational Biology. After careful consideration, we feel that it has merit but does not fully meet PLOS Computational Biology's publication criteria as it currently stands.

While all of the reviewers acknowledge the importance of the methodology, several concerns are raised. In particular, reviewer #3 has serious technical concerns about the method itself, which is the primary focus of this work. These will have to be carefully addressed if the work is to be reconsidered.

Therefore, we invite you to submit a revised version of the manuscript that addresses the points raised during the review process.

We believe that we have appropriately address the comments and have further demonstrated the utility of LEAR. We would like to thank the reviewers for taking the time to make this work even better.

Reviewers' comments:

Reviewer's Responses to Questions
Reviewer #1: The present paper from Bohrer and Larson describes LEAR, a new computational method to reduce the localization error of chromosome tracing experiments, thereby extracting more information.

The is currently great interest in understanding the 3D structure of the genome, its regulation and function. And chromosome tracing is an increasingly common approach to this. Therefore, improving the analysis of chromosome tracing experiments is an important problem, and I believe the LEAR method will be of wide interest in the field.

Beyond the application and description of LEAR, the manuscript also does a great job of educationally discussing the issue of localization error and the consequences (which is poorly understood in the field and the origin of many confusions). The application of LEAR to published datasets is also very helpful, both for the biological insights as well as for illustrating its value.

The LEAR approach appears to me to be sound. I also went to https://github.com/CHB-Bohrer/LEAR/tree/main and although the GitHub repo appears barebones on first encounter, there is a good user guide in the “User_Guide_and_Example_Data.zip” and the Quick User guide seems helpful (the first author even suggests that user text them on the mobile). I think the impact of LEAR will depend on the ease of its use, so making sure that it is very easy to use will be important.

In short, a solid well-written manuscript that should be a wide interest and which seems like a great fit to the journal.

My suggestions are all minor – they do not affect the validity of the approach – but I do think they will be important to address to improve the presentation and balance of the manuscript. Otherwise, I enthusiastically recommend that a suitably revised manuscript be published.

We are glad that the reviewer liked LEAR, and we believe that we have addressed the comments appropriately.

SUGGESTIONS:

1. A discussion of RASER-FISH is missing. Figure 1 in version 1 of the Beckwith 2021 preprint https://www.biorxiv.org/content/10.1101/2021.04.12.439407v1?versioned=true shows very clearly that the chromosome tracing protocol used in all the studies cited in this paper and in all the studies whose data is used here, results in severe perturbations. It is also not surprising. If you boil cells in 50% formamide, it would be surprising if chromatin did not move at least a few tens of nanometers. But it is important to mention this explicitly. I would like to ask the authors to include a brief discussion about de-naturing vs. non-denaturing FISH and the perturbations that results from the standard chromosome tracing procedures. Of course, this is not the authors fault – they are just provided an improved algorithm. But essential to discuss.

We have now included RASER-FISH and mention the additional errors caused by denaturing chromatin tracing methods. We do this in two main places within the manuscription:

matin tracing (63, 67). While there is an inherent uncertainty with all fluorescence imaging techniques, additional error stems from factors specific to the chromatin tracing approach (63). For example, traditional denaturing chromosome tracing protocols utilizing high temperatures and high formamide concentrations, can perturb the natural chromatin by tens of nanometers (6), even despite recent improvements (6, 7). Still, reported errors range from 25-100nm for chromatin tracing

Several sources of error likely still obscure the correlation between enhancer-promoter (E-P) proximity and nascent transcription. Although we enriched our dataset for traces temporally closer to transcription initiation, the fixed-cell nature of chromatin tracing limits temporal resolution, potentially masking underlying relationships (14). Fixation protocols, similar to those observed in traditional denaturing chromosome tracing methods can disrupt natural chromatin organization (6, 7, 62). Moreover, accurately assigning localizations to the correct trace remains challenging, and misassignment would clearly distort quantified E-P relationships (42). For instance, extra localizations in the same genomic region—arising from sister chromatids, aneuploidy, or imaging noise—complicate trace assignment (42). Finally, the uneven stochastic nature of probes labelling a specific chromatin region introduces even more uncertainty, and the field has yet to address the fact that a localized segment of chromatin is not truly a fluorescent point source. Thus, just as LEAR enhanced the correlation between E-P proximity and nascent transcription, addressing these other error sources will likely further resolve discrepancies.

2. Building on that, I would like to ask the authors to include a paragraph on chromosome tracing experiment limitations. First one is cell fixation. Second one is denaturing the DNA, by boiling the cells. Beckwith-Ellenberg has clearly shown this. In an ideal work, the authors would also analyze a non-denatured dataset (e.g. from Ellenberg lab) but this likely be beyond the scope of this paper. Third one is sister chromatids. Since most chromosome tracing experiments are done on cycling cells, on average, about half of all chromosomes will be replicated and appear not as a single chromosome but as a pair of sister chromatids. What is then imaged in the chromosome tracing experiment is the average of the 2 sisters, i.e. the average position of the same DNA region in the 2 sisters. Because replicated chromosomes give 2x the signal (after all, they are replicated) any procedure that removes bad data will further enrich for replicated sisters. Accordingly, chromosome tracing experiments are not really tracing single chromosomes. They are reporting average positions between 2 sisters. This is a major source of error, and more or less all chromosome tracing papers sweep this issue under the rug to my knowledge. There are other issues too. I know this is somewhat unrelated to LEAR and LEAR is great, but it will be important to discuss explicitly in a paragraph in the paper for the audience to appreciate other error sources. It is also important to the key question of the correlation between E-P and txn – the authors nicely show that lower error allows making the connection. If these other sources got fixed, the correlation would likely be even stronger.

We agree with the reviewer that there are many other experimental sources of error that should become common knowledge within the field. We agree that more explicitly discussing these other sources of error

may be beneficial to the readers. We have consequently added the following paragraph to the discussion, which is also included within the response to the first comment.

Several sources of error likely still obscure the correlation between enhancer-promoter (E-P) proximity and nascent transcription. Although we enriched our dataset for traces temporally closer to transcription initiation, the fixed-cell nature of chromatin tracing limits temporal resolution, potentially masking underlying relationships (14). Fixation protocols, similar to those observed in traditional denaturing chromosome tracing methods can disrupt natural chromatin organization (6, 7, 62). Moreover, accurately assigning localizations to the correct trace remains challenging, and misassignment would clearly distort quantified E-P relationships (42). For instance, extra localizations in the same genomic region—arising from sister chromatids, aneuploidy, or imaging noise—complicate trace assignment (42). Finally, the uneven stochastic nature of probes labelling a specific chromatin region introduces even more uncertainty, and the field has yet to address the fact that a localized segment of chromatin is not truly a fluorescent point source. Thus, just as LEAR enhanced the correlation between E-P proximity and nascent transcription, addressing these other error sources will likely further resolve discrepancies.

3. Line 24 cites nice chromosome tracing papers from Nollmann, Boettiger, Zhuang labs, who have done important early work. But there are many many other labs. Chao-Ting Wu did most of the early pioneering work that formed the foundation for the Zhuang work, but many others come to mind like Jan Ellenberg, S Wang etc. Feels slightly unbalanced.

We have added several publications to the list.

4. This is subjective and minor, but I found the 'dynamics' terminology used to be a little odd at times. The first sentence of the abstract says "spatial dynamics". The fourth sentence "displacements". These are terms use for tracking experiments like live-cell single particle tracking. But the point of chromosome tracing is that the cells are dead. And static. I suggest to use static terminology for static experiments, and not terms like dynamics and displacement.

We have changed the word dynamics but have decided to keep the word displacements as we believe it is still the best word to describe the inner workings of LEAR. We also got rid of the "in living" in the second sentence of the abstract to make it clearer that chromatin tracing is a fixed cell methodology.

5. Small thing but x-axis of Fig S7E should go from 0-100 if it is in %, or did I miss something?

Sorry about that, we have changed the x-axis accordingly in Fig. S7E and also in Fig. S5E and Fig. S8E.

6. It would be helpful to state all the assumptions of the method in one place in the main text. Line S32 in the SI says error need not be Gaussian, but other places say it is assumed to be Gaussian.

We extensively describe these assumptions within the revised SI in the "approximating the probability distributions for the goal variances" section. Also, to clear up the Gaussian assumption a bit more we have extensively added to the SI, for example:

the variance of the true position of locus α relative to the observed position of locus β. Because this variance is computed from many datapoints (traces), its sampling distribution can be approximated by a Gaussian via the central limit theorem, provided only that the underlying variables have finite variance. This approximation does *not* require the localization error to be normally distributed.

The mean of this Gaussian is given by

$$E[\mathrm{Var}(T^{\alpha} - O^{\beta})] = \mathrm{Var}(O^{\alpha} - O^{\beta}) - \mathrm{Var}(\epsilon^{\alpha}),$$

as described in the main text. Importantly, this expression is distribution-free: it does not depend on the error distribution, and thus the most critical quantity for LEAR—the mean—can be obtained without assuming normality of the localization error.

Note that we computationally test the application of LEAR with normally distributed localization error. The central assumption of LEAR is that the localization errors are independent and is mentioned in the first section within the main text:

With the experimental data, we can empirically quantify the variance of the differences between a pair of loci ($\ell = \alpha$ and $\ell = \beta$); with the assumption that the localization errors are independent, the variance is the following:

$$Var(O^{\alpha} - O^{\beta}) = Var(T^{\alpha} + \epsilon^{\alpha} - O^{\beta}) = Var(T^{\alpha} - O^{\beta}) + Var(\epsilon^{\alpha}), \quad (1)$$

7. In line 157 they test LEAR on the tracing data of Huang REF34 and add Loc Error to the data, treating the data as ground truth. This felt a little odd to me, because the data is subject to noise and all the other uncertainties of experimental data. Would it not be much better to run a polymer simulations and generate synthetic chromosome tracing data where the ground truth is known, and then add localization error to the polymer simulations? There should be polymer simulation data available for download from various publications using polymer simulations.

The use of polymer simulations was a comment from each of the reviewers. We have therefore added this analysis as an additional figure and section (Case3). The results are mainly the same, but we also demonstrate the utility of LEAR with a smaller localization error of 25nm.

8. LEAR does seem very clear and impressive. But line 211 says “greatly” and then line 214 says “10-20% improvement”, so greatly seems a little overstated.

The previous line 211 was referring to improvements in contact frequency calculations.

9. For the discussion around lines 218-224, the clearest demonstration that there is a quantitative relationship between “contact” and transcription to my knowledge is Fig 4D in Kim-2024 https://www.cell.com/molecular-cell/fulltext/S1097-2765(24)00126-6 which the authors could also discuss here.

We now include this paper (46) but have decided to place it within the introduction, as the discussion around the prior 218-224 was solely focused on the Sox2 system.

10. Very subjective, but I do not love “contact frequency” to be equated with “<200 nm threshold”. Contact to me has mechanistic meanings, the two pieces of DNA are in physical contact. A semi-arbitrary distance threshold is a common method of analysis, but in my subjective view, it introduces confusion to equate a threshold to a mechanism. I would suggest to just call thresholds thresholds, and not imbue them with mechanistic meaning.

We understand that this is a point of contention within the field and that understanding what a ‘contact-frequency’ means with the different methodologies is still unclear to an extent; micro-C contact frequency vs Hi-C contact frequency, etc. But we believe that including the ‘contact frequency’ term is the best for the readership if we clearly define what we mean. To make this clearer to the readership we have added the following:

onic stem cells (mESC). The contact frequency quantified from $\approx$ 1400 traces with a distance threshold of 150nm is shown in Fig. 3A; loci lacking a sufficient number of datapoints were excluded. It is important to state that for chromatin tracing *contact frequency* refers to the proportion of traces where two or more genomic loci are spatially within a defined distance threshold (e.g., 150 nm or 200 nm), which may be different from the contact frequencies determined with other experimental methodologies. The idea was that the empirical data would be treated as the ground truth and different amounts of local- [illegible]

11. Line 290: makes sense since loop extrusion is at its core a 1D mechanism.
Agreed!

12. Fig 5A color bar, ticks are missing a label.
Fixed.

13. Line 318, "inversely proportional to the distances between loci" – can you state whether this is distance in nm or kb.
We have added 'nm' to the main text. Also note that we discuss this result in multiple parts of the manuscript.

14. Line 362-364, yes, but also boiling cells, fixation artifacts, sister replication, etc.

We have discussed the other sources of error throughout, and have modified the text to be consistent:

The application of advanced microscopy methods will undoubtedly continue to push the resolution limits of chromatin tracing, and LEAR will still have utility in certain contexts. Even with the advances of non-denaturing chromatin tracing (6, 7), localization error still stems from two central sources: (1) a limited number of photons and (2) the fact that a localized

15. MINFLUX is awesome but is unlikely to take over any time soon due to practical limitations. I almost feel like the authors undersell LEAR here.

Reviewer #2: Bohrer and Larson introduce a post-processing computational approach to minimize the contributions of measurement error. This work offers the first systematic application of measurement error quantification (already systematically included in many datasets of this sort), to improve the quality and interpretation of the data. Current approaches by contrast have largely relied on these error measurements as control data. Leveraging this information, the approach also offers the first (to my knowledge) attempt at systematic error correction based on a minimal set of assumptions. Overall, the manuscript is well written and represents an important advance in a rapidly growing field. I would support publication, though I recommend the authors consider the issues I discuss below in a revised manuscript.

The iPython notebook runs on the included demo data without problem and produces the expected results. The software and demo data were easy to download and the demo easy to navigate. These are critical features for a software paper and a disappointing number of articles reach this standard on the first go so I commend the authors there.

My primary recommendation would be to test the method on some fully simulated data, which may help provide a better estimate of the maximum improvements that may be achieved by the method (given the logical arguments the authors present about why existing data may underestimate the approaches performance), as well as better identify potential additional artifacts the method might introduce – see below for details. I include several other suggestions for improving the clarity and potentially the rigor of the analyses below, and I apologize in advance for the lack of brevity in these comments, I lacked the time to write a shorter letter.

We thank the reviewer and are glad the software worked out! We address the comments below. We now include a section where we apply LEAR to polymer simulation data showing a similar result.

Primary recommendations:

I think one fully-simulation based dataset + simulated noisy acquisition + method application would be interesting to test the upper limits of the correction, and to identify potential sources of error introduced by the method. I appreciate the power of adding noise to current data as a way of keeping a truly realistic background model, I would not suggest removing that. But there is still much more uncertainty in understanding which features of the data are true variation and which are arising from the noise when using real data. For example, even in the original filtering steps, based-off analyses of the replicate data, the simulations will show what sort of errors are introduced and what frequency by hard thresholds (below the maximum stretch length for the underlying polymer) for the max distance to neighbors. Further, the authors identify a series of quite logical arguments why the true improvement achieved by the method may be greater than those estimated by comparing real data vs real data+ noise or by comparing two noisy replicates to each other. This 'true improvement' (or at least a better understanding of the maximum potential improvement), could be better understood and quantified with a fully known, simulated ground truth.There are numerous tools available for doing polymer simulations these days, I might recommend some some of the loop extrusion simulations from the Mirny lab or other open2c/polychrom contributors.

This recommendation was made by all three reviewers. Consequently, we have added a Case 3 section where we utilize polymer simulations. We show similar results and further demonstrate the utility of LEAR with an even smaller localization error. We have also added a figure.

Figure 2 I think could use some improvement, as I missed the message of this figure on the first read and was going to suggest moving the entire thing to supplement. I think instead some reorganization and modification to the plots would make the message clearer.

2.1 In (iii), is "relative error (STD)" = error with LEAR-corrected data / error of orig data? If so I would label it as something such, maybe make it on log2 axes and maybe leave the above zero part of the graph blank so that it is more apparent at a glance that the method reduces the error, particularly improving Z. Currently I have to stare quite long to figure out what's going on in this figure, and closely examine the tiny numerical values on the y-axis to tell that any improvement has occurred.

We have now modified the figure incorporating your suggestions. It is indeed clearer on the log2 scale. See new figure 2.

2.2 It would also be instructive I think to see the distance maps (or contact maps) before and after the method. I'd be curious if the correlation of the corresponding contact-frequency maps with bulk Hi-C changed at all as well in the before vs. after the correction. As long as the Hi-C data isn't referenced in whatever correction approach is being used (as is the case here), it is a nice orthogonal way to cross-validate the data and validate the correction.

We have decided that utilizing a comparison with Hi-C data is not best for the current manuscript for a number of reasons. The central reason being that it is unclear which distance threshold one should use when comparing the Hi-C and chromatin tracing data. It brings up the possibility that a distance threshold of 100nm may perform better when LEAR is applied but a distance threshold of 350nm leads to a better performance with the raw unprocessed data. We agree that it can be a nice orthogonal method to validate but feel it best not to include it here due to different thresholds leading to differences in performance.

2.3 The panels (ii) are a bit of technical detail specific to the datasets (what their actual errors were before and after, I also can't tell from the text if this is before or after applying the method). Maybe these could go into a supplement to make room for some more maps.

This point was addressed in original lines: 128-136. We believe that it is an important point highlighted in panels (ii). Here we show that the localization errors of a locus can be different between initial images and repeat images within the same cell. This error is purely for the raw data and without the application of LEAR. For example, in the Su et al. dataset of Fig. 3Cii, the X localization error of the initially imaged is around 50nm, but upon re-imaging the same locus (in order to actually determine the localization error), the localization error is worse, being around 75nm. We describe the process we used to determine these values in the SI. We have added an extra sentence at the end of the referenced paragraph to more firmly highlight this result and explicitly state the connection to the raw data. We hope that this change clears things up.

regarding the actual resolution of an experiment — the localization error is apparently not accurately quantified using only the distances between initially imaged and repeat imaged loci. Simply put, for these chromatin tracing protocols, repeatably imaging the same locus can result in a different localization error than when the locus was initially imaged.

2.4 On first read I got more out of Fig S2, which shows how different data properties (like number of loci, detection efficiency, sigma-guess) effect the 'relative error'. After deeper study I think Fig 2 panel (iii) have important data relevant to the main claims of the paper, but (i) and (ii) are less informative that the graphs in Fig S2. The authors might consider moving some of these data up to the main Figure.

We are glad that the referee finds that SI figure informative. However, we believe it best to keep the empirical data together and the simulation data separate.

Minor recommendations:

How much of the effect of LEAR shown in Fig 3 and 4 is explained by simply reducing the 3D distances through the 'correction' process. The increased pairwise and 3-way contact frequency after error addition in Fig 3 H and I with a fixed contact threshold is also expected if one isotropically expands 'correct' data. Similarly with the increase in contact frequency in the Huang data with and without the CTCF insertion. Do the average 3D distances get smaller? If so by how much? I appreciate a compaction effect wouldn't explain all of the improvements (such as those shown in fig 2, or increasing the measured strength of insulation), but some supplement on the effect on total average distance (e.g. radius of gyration) and the contribution of any systematic change in this average to the changes in contact threshold could be addressed.

With the removal of localization error, yes, the 3D distances get smaller, but mainly for loci in close spatial proximity. This effect is seen with LEAR and was first systematically investigated by Churchman in 2006 PMID: 16258038. The mean distances between loci that are separated by a distance much larger than the scale of the localization error do not really change, similarly for very small contact frequencies. Thus, if the reviewer is suggesting to 'compact' the chromatin tracing data at the trace level and see if this improves the localization error similar to LEAR, we suspect this transformation would not work. A simple compaction would cause the mean distances between spatially distant loci to also change, which would be quite different from correcting localization error which works at short distance.

Multi-way contacts +/- cohesin

With LEAR correction, can you see an effect on cooperative contacts at CTCF sites (similar to that reported by Hafner et al 2023 (Hafner et al. 2023), and multi-contact 4C (Allahyar et al. 2018))

Based on data shown in the original Fig. 5C+D, you can see that all possible 3-way contacts follow the same curve. Therefore, within this dataset, CTCF sites show cooperativity to the same extent given their increased 2-way contact frequencies. So, one way to think about this is if CTCF/Loop extrusion leads to an increase in 2-way contact frequencies (naturally increasing multi-way contact frequencies), the

cooperativity then leads to an even further increase in the multi-way contact frequencies, for they are sensitive to the underlying two-way contact frequencies.

Within Hafner et al. they saw 3-way contacts above that expected from the two-way contacts only utilizing the untreated cells. Then they compared how the 3-way contacts varied between conditions, not explicitly looking at the cooperativity in that case, making it a little difficult to compare exactly with our findings here. Allahyar et al., did not utilize chromatin tracing, so we would not directly compare here either.

The subsection title is a bit problematic - it would read better as ‘not the only mechanism’ rather than ‘not the mechanism’. In addition to being overstated, the ‘not the mechanism’ comment is very much in line with the picture presented by the Bintu, Mateo, 2018 data, which shows little change in the ‘cooperative’ 3-way contacts upon cohesin degradation (though I appreciate the additional quantification approach used here). The 4-way interaction seen after the correction, for example, is more novel.

We have changed the subsection title to the following: Cooperativity in multi-way contacts is not explained solely by loop extrusion.

The discussion of this result (in the main text, before the discussion section) seems a little under-developed. It is unclear if the authors are concluding that the effect on 4-way contacts is purely an effect of increased 2-way contacts, or if they are suggesting that an inherent proximity-dependent cooperativity among chromatin contacts is amplified by the contact.

We did not actually investigate the relationship between 4-way contacts and the underlying 2-way contact frequencies in the original submission. It is an interesting question, and we should have addressed this point. We have added a new figure within the Supporting Material showing this relationship, indeed suggesting that the cooperativity is similarly dependent upon the two-way contacts just as the 3-way contacts are. We have added the following text:

followed the same curve both without (Fig. 6C) and with LEAR (Fig. 6D). Probing further, we performed the same analysis but for 4-way contact frequencies and obtained a similar result (Fig. S10). These outcomes are significant because it indicates that the cooperative behavior primarily hinges on the frequencies of two-way contact frequencies. Put another way,

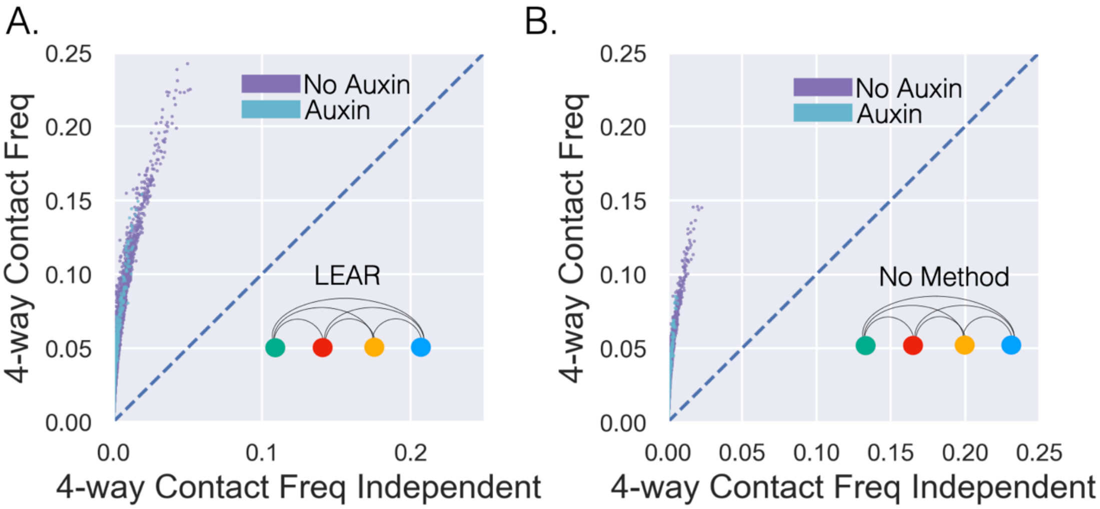

Figure S10: *Cooperativity in 4-way contacts is not dependent upon loop extrusion:* A) The 4-way contacts compared to the expected 4-way contact frequency if the 2-way contact frequencies contributed independently, with and without auxin with the application of LEAR. B) The same as *A* but without the methodology.

Fig S6 – The layout could be improved. The regions in A and C could be shown as zoom ins on E so that the comparison would be easier for the reader. Especially lacking axis labels in E it is hard for those not familiar with the size and coordinates to match these up.

We have changed the figure so that the regions in the other figures can be adequately seen in E.

Data filtering

While a very elegant argument is outlined on why some data points are erroneous (those that were repeat labeled and gave two very different answers, or those that were repeat labeled and gave distances far from the nearest neighbors both times, the authors filtering solution to address this is rather buried in the supplement, and appears to be a bit simplistic (which is certainly meritorious in easy-to-apply, but maybe could be made a little more precise). On one hand I thought this was an important insight that merited a clearer explanation and discussion in the main text (just a couple more sentences would go a long way). On the other hand, I think a few more controls in the supplement and a more algorithmic way of settling on the final cut-off (or an alternative to what I understood as a simple hard threshold) would also be nice.

We too agree that this is an important finding. To more clearly highlight that there are erroneous datapoints, and that future investigations should look further into this problem, we have added a bit to the main text, see below. We also believe that there is likely a better way to filter out bad localizations but to address this problem properly it would be beyond the scope of this work. We address the other comments below.

localizations depending upon how the observed localizations deviated from a specific model (38, 42). However, little evidence was given to justify the discarding of certain localizations beyond a better agreement with chromosome capture contact frequencies (42). Importantly, we provide multiple lines of evidence justifying 'filtering' within the Supporting Material Section 8. A more complicated filtering approach rooted in polymer theory could be further developed taking into consideration the nature of off target localizations quantified here (42). We must also state that due to this problem, the error improvement from LEAR is likely underestimated (Supporting Material Section 8.2). Throughout we only tested the application of LEAR

4.2 Additionally, it was unclear how exactly the thresholds were applied. There appear to be hard cut-offs: 500 nm (or 400 nm) in the nearest neighbor jump – what should one do about points with missing data? E.g. 2 points missing between, allow 1000 nm jumps?

Sorry, this information was missing in the initial draft. We now describe how we handled missing values within the supporting material.

Specifics of Simple Filtering Algorithm

Here we describe the simple filtering algorithm that we applied to the chromatin tracing datasets. To remove unreliable localizations from chromatin tracing data, we applied a nearest-neighbor distance filter to each trajectory. For each locus within a trace, we computed the coordinate-wise separation (X, Y, Z) from its immediate upstream and downstream loci. In cases where neighboring positions were missing, distances to loci two positions away were also considered. The minimum distance to neighboring loci was then calculated independently for each coordinate axis. Localizations were excluded if the minimum separation along any axis exceeded a set distance threshold specific for the dataset or if no single neighbor could be identified.

4.3 Can one do better than a hard cutoff? Based on polymer theory, the distance between two points have heavy tailed distributions with rather long tails. The precise shape is affected by details of the theory, e.g. crumpled/unknotted, vs. equilibrium globule vs. dilute/Rouse polymer – but all have finite support (e.g. a hard maximum cut-off at the truly stretched polymer). Notably this polymer stretched distance: 50 kb at 10 nm/200 bp = 50 nm per kb = 5000 um truly stretched, so achieving 1000 nm end-end is not too much of a stretch, though empirical data show this is very unlikely. I think the physically realistic hard-cut off at some estimate of the maximum stretch of the polymer is too lenient, but a truly hard-cutoff is rather non-physical (i.e. it would exclude some error-free data), though likely not exclude enough data to make an practical difference. Can one do some probabilistic rejection of data based in its observed vs. expected frequency from a polymer model?

We believe that one could for sure do better than a simple hard cutoff. And the methodology that comes to mind is that of Jia et al. 2023 Nat Biotechnol. Again, while we believe it is beyond the scope of the LEAR manuscript here, one could utilize the polymer approach of Jia (deciding whether to discard a localization), rooting it in experiment / strong priors using a similar approach to the one we used.

4.4 I don't think it's necessary for the authors to be apologetic about the need for filtering, though I very much appreciated the analysis of the justification for filtering. I think this could fairly be identified as an equally important step as "denoising" that is achieved by the core LEAR algorithm, and a subject matter in need of more rigorous intellectual engagement in the community, which has previously been handled on a very ad hoc basis. It could be instructive also to compare prior ad hoc decisions to the more data-driven, systematic approach used here. For example, prior work using the Bintu, Mateo, 2018 (Bintu et al. 2018) data vary dramatically in which traces they actually use: (Goundaroulis et al. 2019) toss all but 243 of the over 4,500 traces when using the data to say something about knots in the path. By contrast (Conte et al. 2020) toss way fewer traces from these data using a different set of logical sounding but equally ad hoc criteria: "Here and in the following analyses, we filtered out the experimental single-cell distance matrices having NaN values for more than 80% of the entries and, in order to remove outliers, the matrices having a Pearson correlation <0.01 with the others are also removed in both models and experiment" These data admittedly contain some dubious long range jumps. However, these jumps are not so long as to be

categorically ruled out on simple physical properties, such as the maximum stretch distance of DNA however, nor are they filtered by brightness. Comparison to Hi-C provides an orthogonal measure that indicates some barcode positions appear to be noise uncorrelated with the orthogonal approach, whereas most of the data correlate strongly (and exhibit other properties expected of a polymer, such as the preference to be nearer in 3D space to sequences known to be closer in sequence space along the polymer). It's less clear that there's any physical justification for rejecting outliers based on Pearson correlation or % missing data (missing data is likely a stochastic event driven by stochastic properties of the a labeling approach that relies on chance to reduce a duplex strand of DNA to a single copy by randomly chopping and melting out bits, hoping that 1 is removed not both). Such a process is not expected to give uniform coverage in each trace, nor are data from higher-miss-fraction traces necessarily an 'worse' a measure of reality.

In contrast to unjustified ad hoc approaches, these authors provide a more rationale, data driven explanation of which data to drop. This important contribution could be expanded, and maybe some more general guidelines could be derived from this analysis for future work. Such guidelines could also be used in iterative fitting algorithms – in practice multiple potential 'spots' can be detected in a single image and the software could use some guide on which ones to choose (e.g. brightest is not always best).

We most definitely agree that a more rigorous investigation should be done, and are glad that the reviewer appreciates our data-driven justification for excluding data. We were not aware of the other ad hoc specifics of excluding data. While we share the same passion as the reviewer here, we do believe that this is beyond the scope of this work and should likely be a separate publication in its own.

Minor Text issues to clarify

"the number of loci that were repeatably imaged, with the 50kb resolution data having the most repeatably imaged loci ≈ 20"

I think some further unpacking of what is meant by "the number of loci that were repeatably imaged" is necessary. I suspect many readers will mistakenly interpret this as the number of loci that could be reliably detected across cells. I believe what the authors mean is the number of loci that were imaged multiple times within each cell for the purpose of quantifying measurement error.

The reviewer is correct in what we meant to convey, and we agree that some of the readers may get confused. We have modified the text in the following way to make it clearer:

> varied as well as the number of loci that were repeatably imaged, with the 50kb resolution data having the most repeatably imaged loci ≈ 20; these are loci that were repeatedly imaged in each cell specifically to quantify the localization error. The chromatin organizations of these datasets greatly varied, with the 2kb resolution data showing the smallest distances overall

[intro] ". While there is an inherent uncertainty within all fluorescence imaging techniques interrogating spatial features less than the wavelength of visible light,"

I'd reword this. There is extensive confusion in the scientific community about the relation of the diffraction limit and multiplexed imaging - with many scientists incorrectly associating the Abbe diffraction limit with ability to resolve two fluorescently labeled spots, labeled with chromatically distinct fluorophores or in sequential rounds with same fluorophore. As the current authors I am sure appreciate, if two elements of DNA are labeled with fluorophores of the same wavelength and imaged at the same time, there is indeed a diffraction limited resolution problem. If the fluors are different colors (or in different rounds) the *wavelength of visible light* has a minimal effect on resolution that is almost always dominated by 1 of dozens of other sources of uncertainty. It would be equally correct to write that STORM and PALM are

limited by the wavelength of light. While technically true, this would be unnecessarily confusing to the reader, as the wavelength limit is too intricately connected with the idea of the diffraction limit, and as other sources -(quantum efficiency, photon shot noise, duty cycle) typically dominate the contributions of the wavelength of light in chromatin tracing data (as they also tend dominate most STORM and PALM data and related SMLM techniques).

We modified the text to be the following:

However, localization error in fluorescence imaging sets a limit to accuracy which becomes uniquely challenging with chromatin tracing (63, 67). While there is an inherent uncertainty with all fluorescence imaging techniques, additional error stems from factors specific to the chromatin tracing approach (63). For example, traditional denaturing chromosome tracing protocols utilizing high temperatures and high formamide concentrations, can perturb the natural chromatin by tens of nanometers (6), even despite recent improvements (6, 7). Still, reported errors range from 25-100nm for chromatin tracing

For what it's worth: I'm pretty convinced the primary limitation to resolution in chromatin tracing comes from the size of the DNA labeled in most data sets. This region is 2 kb in the highest resolution traces, and more often 10 kb or 50 kb in much of the high quality public data. This distance is not a point source, and at ~ 10 nm/nucleosome, the finite size occupied by a flexible chromatin polymer of 10-250 nucleosomes can easily be on the scale of the distances measured in the microscopy (50-1000 nm is a typical range, and 2 kb can be 100 nm and 50 kb can be 1000 nm though it averages closer to 150 nm).

The labeling procedure is also not uniform, since it relies on stochastic elimination of DNA to produce single-stranded DNA for binding (transient DNA melting is insufficient for labeling, as the complementary strand is longer than the probe, it can always undergo a toe-hold mediated strand-displacement reaction to evict a fluorescent oligo, unless it is actually cleaved and released from the cell or degraded). The data analyzed here was all created using acid and heat denaturation, which I believe stochastically nicks the DNA backbone allows short strands to drift away. Consequently a labeled 10 kb region will consist of variably sized lengths of actually exposed target (including some cells with no exposed target, hence the drop-out rate, even when imaged in thin sections with cameras that have no difficulty detecting single cy5 fluors, the detection efficiency for short regions is never 100%, and it increases with longer regions as the probability of retaining the target DNA so there's something for the oligo to label in the first place increases). This stochastic variation in the size and position of what is labeled, cell-to-cell, further contributes to an uncertainty in the interpretation of the population level distribution of the 3D distance between elements. – The elements themselves actually have some positional variation due to the stochastic labeling and these labels are distributed over a polymer of finite size, not a point source. For some datasets, drift correction can be a significant limitation to resolution as well - many studies use beads to track the position of the coverglass to correct stage drift, but this contains no information about minor changes in the position of individual cells (or individual chromosomes), which may change subtly over days of repeated washes and imaging. Most approaches use z-scans, and the return accuracy and alignment of z-position does not exceed 50 nm precision on all systems unless some explicit care is taken. For computational efficiency, rounding some analyses rely on image based correlation analysis to correct drift, in which images are aligned on finite sub-pixel shifts (e.g. 25 nm in Mateo 2019), setting another hard-limit to resolution accuracy.

We really appreciate this excellent insight. While we initially discussed the problem of the chromatin not being a point source in the original draft, we now include a paragraph discussing more sources of error that will hopefully inform the readership just how big of a problem 'error' actually is within chromatin tracing:

Several sources of error likely still obscure the correlation between enhancer-promoter (E-P) proximity and nascent transcription. Although we enriched our dataset for traces temporally closer to transcription initiation, the fixed-cell nature of chromatin tracing limits temporal resolution, potentially masking underlying relationships (14). Fixation protocols, similar to those observed in traditional denaturing chromosome tracing methods can disrupt natural chromatin organization (6, 7, 62). Moreover, accurately assigning localizations to the correct trace remains challenging, and misassignment would clearly distort quantified E-P relationships (42). For instance, extra localizations in the same genomic region—arising from sister chromatids, aneuploidy, or imaging noise—complicate trace assignment (42). Finally, the uneven stochastic nature of probes labelling a specific chromatin region introduces even more uncertainty, and the field has yet to address the fact that a localized segment of chromatin is not truly a fluorescent point source. Thus, just as LEAR enhanced the correlation between E-P proximity and nascent transcription, addressing these other error sources will likely further resolve discrepancies.

4. [abstract]: "we then applied our approach to existing chromatin tracing data that probed the relation between chromatin organization and Sox2 regulation, where previous work found no correlation between enhancer-promoter proximity and transcription bursts in individual cells." See ALSO: [intro] "no direct relation to nascent activity was found in individual cells"

I think this is an overstatement, I believe there is a correlation in these data without correction (from our own plotting of these data without correction, as well as based on statements in the original article).

Specifically:

"the median enhancer–promoter distances at the Sox2 gene with coincident nascent transcripts were slightly but significantly shorter than those on the resting loci (Extended Data Fig. 10c,d)." - see ED Fig 10d of ref 32.

"These results, taken together, suggest that enhancer proximity is positively correlated to transcriptional activity at the target gene" (ref 32)

While I believe the original data showing the (weak yet statistically significant) correlation could have been shown and explained better, I think it is inappropriate for the current work to say no correlation was found or to conclude no direct relation to nascent activity in individual cells was found. I don't have a problem with the authors saying the relationship was previously "obscured by localization error", (line 67) to me this doesn't imply it was totally invisible and concluded not to exist. I'd recommend a phrasing of this sort also be adopted for the abstract.

We understand what the reviewer is saying here, and it is true that there was a weak change in the median distance. Note that the authors also found that there was no change in bursting probability as a function of enhancer-promoter distance threshold, which we believe is a similar measure. We have changed the writing within the manuscript to address this concern. The main modification of the text is shown here:

the super-enhancer and promoter in single cells (3, 38, 57), raising questions about the relevance of E-P spatial proximity and transcription regulation. Specifically, live-cell microscopy has shown no relation between E-P distance and nascent transcription (3, 57). However, chromatin tracing has resulted in conflicting results (38); a weak but significant change in the median E-P distance with nascent transcription was observed (a change of $\approx$ 18nm), but there was no clear difference in the proportion of alleles showing active transcription as a function of E-P distance threshold. We therefore sought to use the

5. Typo: Line 340, "Interestingly, due to the cooperatively, higher-order multi-way contacts could be essentially…" should say "cooperativity"
Thanks, we have fixed this text.

Reviewer #3: This article by Bohrer and Larson introduces a new methodology, LEAR, to reduce localization error in chromatin tracing experiments (i.e. collections of 3D conformations of a portion of a chromosome, obtained by sequential DNA FISH microscopy). This approach relies on an estimation of the localization error and on the realization that, for each imaged DNA locus, its pairwise distance distributions with all the other DNA loci in the collection of conformations bear information that can be harnessed to reduce localization error effects. Using publicly available datasets, the authors evaluate how LEAR performs at restoring artificially-deteriorated experimental data and then revisit questions about enhancer-promoter contacts at the Sox2 locus and multi-way chromatin contacts with and without the cohesin complex.

I find the motivation strong and the approach original, but I have several concerns — especially about the interpretability of the "corrected" data, the limited value of the validation strategy, and the clarity of the manuscript. Below I outline the most important points. The application to experimental data illustrates the method but yields modest biological insight.

*** Major comments ***

1. A central concern is that the method cannot meaningfully correct individual traces. For each locus α, the method estimates the N correction values based on only L−1 constraints (i.e. vector C^α, deduced from $Var(T^\alpha-O^l)$ for all the loci l other than α). Since N >> L in typical datasets, the problem is severely under-constrained. The manuscript gives the impression that individual corrected traces A^α are meaningful, which they are not. Instead, the only meaningful outputs are ensemble-level statistics, whether they are pairwise (contact or distance matrices) or higher-order (e.g. 3-way) statistics. At the level of single traces, any additional structure created by the correction procedure is artificial: the true conformation cannot be reconstructed from the available data, and no trace-level information has been gained. It is essential that this be clearly stated to avoid misinterpretation by future users.

We disagree. We demonstrate that the relative localization in Figures 3E and 3G is improved at the individual trace level. This quantity is simply $(A^\alpha-T^\alpha)/(O^\alpha-T^\alpha)$, which is the relative amount of error for locus alpha, which we show decreases to varying degrees with the use of LEAR. Moreover, while it is clear that the LEAR improvement varies based on the specific experimental system, our analysis suggests that there is a worthwhile improvement for the variety of experimental datasets that we utilized. Still, to make the point clear, and building off of point #2 below utilizing polymer simulation data, we show the mean error per trace before and after the application of LEAR. This information is similar to relative error per locus, and we believe these results demonstrate that LEAR improves the localization error at the individual trace level. The relative localization error per trace is now shown in the new Fig. 4E.

2. The part on "simulated data" is unconvincing. The authors do not generate synthetic data with known structures. Instead, they take experimental data, apply LEAR once and treat it as "ground truth," then add noise and check whether LEAR can subtract it back. This is a peculiar way to test a method. It lacks realism and fails to assess whether LEAR can recover biologically meaningful features. A more convincing validation would involve polymer simulations (e.g. with loop extrusion) or even simple random conformations that contain by construction features such as looped structures or enriched 3-way contacts.

We have now applied LEAR to polymer simulation data. This analysis is described in a new section within the manuscript and a new Fig. 4.

3. The article is difficult to navigate due to its structure and referencing. In the main text, figures S4–S8 are mentioned before S1–S2, and S3 is never cited. The "Supporting Material" is often referenced without clear

indication of which section is meant, and within it, forward and backward references are frequent and vague ("see below," "discussed more later"), making it hard to follow. There is no clear way to navigate all this material. I list this as a Major Comment –for a reader trying to understand the method, this maze-like structure is a major obstacle.

We have rearranged the figures so that they are referenced in order. And we have added specific sections to the referenced Supporting Material.

*** Minor comments ***

4. A key limitation is that LEAR can only be applied if $Var(\epsilon^\alpha)$ is known. This requires re-imaged loci (or strong assumptions about what $Var(\epsilon^\alpha)$ would be in an experiment where loci were not re-imaged). The manuscript should make this clear (and from the start) to avoid giving a false sense of general applicability.

Yes, the reviewer is correct. We originally referenced this limitation within the abstract and within the first section of the results. To make this limitation even more clear, we have added the following:

$(Var(\epsilon^\alpha) \to 0)$ the variance above would approach $Var(T^\alpha - O^\beta)$; we refer to this quantity as the 'goal variance' which we can calculate given an approximation of $Var(\epsilon^\alpha)$. We describe a few approaches one can use to estimate $Var(\epsilon^\alpha)$ in specific cases in Supporting Material Section 6, but it is important to state that an estimation may need to be quantified experimentally.

5. Regarding the last analysis of the Bintu et al. datasets, a simple hypothesis to explain the observed correlation between contact frequencies of distinct locus pairs (what the authors call "cooperativity") is global variability over the observed region. For example, cells might be in different states (e.g. cell cycle phases), or the entire region might experience global effects such as changes in nuclear positioning or variations in local nuclear context that affect overall compaction. This or anything that affects the observed region globally would naturally lead to correlated contacts between all locus pairs, which would manifest as elevated 3-way contact probabilities that deviate from expectations assuming independent on 2-way contacts. This simple scenario would indeed be independent of loop extrusion and should be discussed as a plausible model.

Good point. We have added a few sentences to the discussion. Here is the modified text:

tent regardless of whether loop extrusion was eliminated. A plausible explanation for such cooperativity is global variability across the region. For example, shifts in nuclear positioning could affect the overall compaction of the region as a whole and naturally produce elevated 3-way contact probabilities beyond those expected from independent pairwise interactions. However, this mechanism does not currently explain why the cooperative behavior was solely dependent upon the underlying pairwise contact frequencies, and if it is not a result of global changes, it indicates that the underlying mechanism is more dependent upon chromatin affinities. Lastly, if the trend holds, it suggests that chromosomal capture contact frequencies

6. In the paragraph around line 94 of the main text, it would help to clarify that $Var(T^\alpha - O^\beta)$ is discussed as a distribution because of the finite number of traces, i.e., it refers to sample variance. Earlier, the term "variance" referred to population variance. Using this standard terminology (sample variance and population variance), here and in the Supplements, would be preferable and would improve clarity.

The modified Supporting Material now uses this terminology.

7. And using known results about sample variance distributions: To estimate $\sigma_{\alpha,\beta}$, instead of using Algorithm 2, couldn't one use the fact that sample variance follows a chi-squared distribution? That would give $\sigma_{\alpha,\beta} = \mu_{\alpha,\beta} \sqrt{2/(N-1)}$. At the very least, this should be discussed.

We understand what the reviewer is getting at here, but we do not believe the problem results in the sample variance following a chi-squared distribution. Still, we agree with the reviewer that this issue should likely be discussed with more specifics. Consequently, we have modified the Supporting Material to better explain why we used Alg. 2., and why we do not believe it follows a chi-squared distribution. See new section 2 of the Supporting Material.

8. The phrase “we can determine the ground truth” (abstract) seems too strong. Inverse problems by construction only allow us to infer or approximate a ground truth, not determine it.
We have altered the wording.

9. In Equation (2), the “probability” is actually a probability density.
Fixed.

10. Figure 2 would benefit from explicitly stating what “relative error” refers to.
The lack of clarity within Fig. 2 was also mentioned by Reviewer 2. We have modified the figure so that the y-axis label states exactly what it is.

11. In Algorithm 2, it would improve readability if you used the same notation as in the main text: using C^{sim} rather than ϵ^{sim}.
We have modified the Algorithm with your recommendation.

12. For comments, Algorithm 1 uses “%” and Algorithm 2 uses “#”.
We have changed Algorithm 1 to use #.

13. In the section that start on page 4 of the Supporting Material, why "$Contant$"? Did you mean "$Constant$"?
Thanks for catching this, yes, we have changed the Supporting Material to correct this.
